# A survey of factors associated with the successful recognition of agonal breathing and cardiac arrest by 9-1-1 call takers: design and methodology

**DOI:** 10.1186/1471-227X-9-14

**Published:** 2009-07-31

**Authors:** Christian Vaillancourt, Jan L Jensen, Jeremy Grimshaw, Jamie C Brehaut, Manya Charette, Ann Kasaboski, Martin Osmond, George A Wells, Ian G Stiell

**Affiliations:** 1 Ottawa Hospital Research Institute, Clinical Epidemiology Program, The Ottawa Hospital – Civic Campus Floor 6, Box 685, 1053 Carling Avenue, Ottawa Ontario, K1Y 4E9, Canada; 2 Department of Emergency Medicine, University of Ottawa, Ottawa, Canada; 3 Division of Emergency Medical Services, Dalhousie University, and Emergency Health Services, Halifax, Canada; 4 Department of Medicine, University of Ottawa, Ottawa, Canada; 5 Department of Epidemiology and Community Medicine, University of Ottawa, Ottawa, Canada; 6 Department of Pediatrics, University of Ottawa, Ottawa, Canada

## Abstract

**Background:**

Cardiac arrest victims most often collapse at home, where only a modest proportion receives life-saving bystander cardiopulmonary resuscitation. As many as 40% of all sudden cardiac arrest victims have agonal or abnormal breathing in the first minutes following cardiac arrest. 9-1-1 call takers may wrongly interpret agonal breathing as a sign of life, and not initiate telephone cardiopulmonary resuscitation instructions. Improving 9-1-1 call takers' ability to recognize agonal breathing as a sign of cardiac arrest could result in improved bystander cardiopulmonary resuscitation and survival rates for out-of-hospital cardiac arrest victims.

**Methods/Design:**

The overall goal of this study is to design and conduct a survey of 9-1-1 call takers in the province of Ontario to better understand the factors associated with the successful identification of cardiac arrest (including patients with agonal breathing) over the phone, and subsequent administration of cardiopulmonary resuscitation instructions to callers. This study will be conducted in three phases using the Theory of Planned Behaviour. In Phase One, we will conduct semi-structured qualitative interviews with a purposeful selection of 9-1-1 call takers from Ontario, and identify common themes and belief categories. In Phase Two, we will use the qualitative interview results to design and pilot a quantitative survey. In Phase Three, a final version of the quantitative survey will be administered via an electronic medium to all registered call takers in the province of Ontario. We will perform qualitative thematic analysis (Phase One) and regression modelling (Phases Two and Three), to determine direct and indirect relationship of behavioural constructs with intentions to provide cardiopulmonary resuscitation instructions.

**Discussion:**

The results of this study will provide valuable insight into the factors associated with the successful recognition of agonal breathing and cardiac arrest by 9-1-1 call takers. This will guide future interventional studies, which may include continuing education and protocol changes, in order to help increase the number of callers appropriately receiving cardiopulmonary resuscitation instructions, and save the lives of more cardiac arrest victims.

**Trial registration:**

Clinicaltrials.gov NCT00848588

## Background

### Out-of-hospital cardiac arrest

Coronary artery disease is the leading cause of mortality in North America and is the condition that most frequently leads to sudden cardiac arrest [1]. Cardiac arrest refers to the sudden cessation of cardiac mechanical activity as confirmed by the absence of signs of circulation [2]. More than 40% of all deaths from coronary artery disease occur suddenly, and often constitute the victim's first manifestation of heart disease [3]. Sixty-five percent of all cardiac arrests occur outside the hospital setting [4]. The incidence of out-of-hospital cardiac arrest (OOHCA) in Canada is estimated to be 55 per 100,000 population, resulting in more than 17,875 deaths annually [5]. Men and women of all age groups can be affected. Canadian cardiac arrest victims collapse in their own home 85% of the time, and 50% of cardiac arrests are witnessed by a family member or bystander. Less than 20% of all OOHCAs receive bystander cardiopulmonary resuscitation (CPR), and the overall rate of survival to hospital discharge rarely exceeds 5% [5].

### The Chain of Survival

The American Heart Association has developed the "Chain of Survival" to indicate the steps in community response to OOHCA [6]. The four "links" in the chain include: 1) Early Access, 2) Early CPR, 3) Early Defibrillation, and 4) Early Advanced Care. The four components of the Chain of Survival are linked to imply that cardiac arrest care is only as strong as its weakest link. The Ontario Prehospital Advanced Life Support study included more than 10,000 cardiac arrest victims and is the largest multi-center prehospital study on cardiac arrest completed to date [7]. This study confirmed a significant survival benefit from early access to care, early bystander CPR, and early defibrillation, but found no added benefit from early advanced care (advanced airway and drugs).

In the "Early Access" link of the chain, a 9-1-1 caller is rapidly put in communication with a medical dispatch centre. In the case of a medical emergency, such as suspected cardiac arrest, a 9-1-1 call taker will collect information on the nature of the call and dispatch appropriate emergency medical services (EMS) unit(s), while aiding the caller in assisting the victim when possible. In Ontario, 9-1-1 call takers are located across the province in twenty-three medical dispatch centres. Ontario 9-1-1 call takers are not health care professionals and come from various educational backgrounds [8]. They receive six weeks of training with an instructor to learn how to navigate dispatch instructions, followed by a six-month preceptorship [9]. Most Ontario medical dispatch centres use call taking protocols designed and administered by the Ministry of Health and Long Term Care. Two Ontario medical dispatch centres use the Medical Priority Dispatch System [10]. This system is a standardized set of dispatch protocols produced by the National Academy of Emergency Dispatch in the United States. This system is used in 23 countries around the world.

"Early CPR" has been clearly shown to be a factor associated with increased survival – a victim is almost four times more likely to survive a cardiac arrest event when he/she receives bystander CPR [7]. Despite various community interventions in the past, bystander CPR rates remain low in Canada and rarely exceed 15% of all cardiac arrest cases in Ontario [5]. CPR instructions delivered by 9-1-1 call takers have been shown to significantly improve community bystander CPR rates [11-14], and received a Class IIa recommendation from the American Heart Association and the Heart and Stroke Foundation of Canada [15]. In Ontario, 9-1-1 call takers began offering CPR instructions to callers reporting suspected cardiac arrests on April 1, 2004. However, the success of this intervention in increasing bystander CPR rates and ultimately survival to hospital discharge is directly linked to the ability of the call taker to accurately identify cardiac arrest over the telephone [14].

### Call taker identification of OOHCA

In previous studies, the ability of 9-1-1 call takers to accurately identify cardiac arrest has been reported to range from 47% to as high as 90% [16-19]. A recently published study conducted in Ottawa reported similar results: call takers correctly identified 56.3% of cardiac arrests during the first nine-month period following the implementation of assisted CPR instructions [14]. Agonal breathing, often present early in cardiac arrest victims, can wrongly be interpreted as a sign of life by 9-1-1 call takers, and is believed to be a key factor explaining why cardiac arrest is not identified [14].

Agonal breathing is defined as ineffective, gasping respiration occurring early in cardiac arrest [20]. Agonal breathing has been variably described by 9-1-1 callers. Some of the more common descriptions include: barely or occasionally breathing, irregular breathing, laboured breathing, sighing, gurgling, moaning, groaning or snorting [16,21]. Previous observational studies have reported agonal breathing in as many as 55% of witnessed cardiac arrests, however the true incidence is likely higher since establishing the presence or absence of agonal breathing was determined retrospectively and relied solely on the callers' descriptions of breathing during review of 9-1-1 call recordings [14,16,19,21]. Previous research has reported increased survival in patients with agonal respirations when compared with patients without agonal respirations (27% vs. 9%; p < 0.001) [21]. However, agonal breathing is frequently mistaken as a sign of life by 9-1-1 call takers, and represents a significant proportion of missed diagnoses of cardiac arrest – up to 50% in some studies [14,19]. If more cardiac arrest cases can be correctly identified by 9-1-1 call takers, there is the potential to increase the proportion of victims receiving early bystander CPR, and ultimately improve survival for out-of-hospital cardiac arrest.

### The Theory of Planned Behaviour

The Theory of Planned Behaviour (TPB) can be a useful, systematic approach to identify barriers to and facilitators of change, and aid in the design of appropriate forms of intervention. [22-29]. The TPB proposes that the strength of an individual's intention or motivation to engage in a behaviour, and the degree of control they feel they have over that behaviour (perceived behavioural control) are the proximal determinants of engaging in that behaviour [30]. This model has been used to study health behaviours of patients and individuals as well as the actions of health care workers, with over 800 published reports utilizing the method [31].

**Figure 1:**
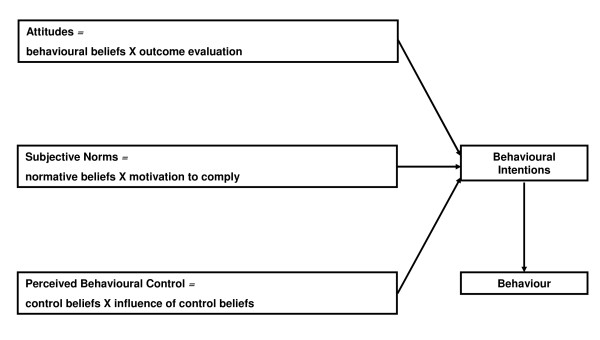
**Constructs of the Theory of Planned Behaviour**. Adapted from [22,31].

The TPB also proposes that intention strength is determined by three variables: attitudes towards the behaviour (determined by beliefs about the consequences of the behaviour and perceived importance of those consequences), subjective norms (a product of perceptions of the views of other individuals or groups about the behaviour, and the strength of the individual's desire to gain approval of these groups) and perceived behavioural control (a function of beliefs about factors likely to facilitate or inhibit the behaviour – these might include organizational constraints and patient/caller preferences). See Figure 1. The TPB states that a single behaviour should be studied and explicitly described in terms of its target, action, context and timelines [32]. We propose to apply the TPB to study 9-1-1 call takers' motivation with respect to the identification of cardiac arrest victims over the phone and administration of CPR instructions (behaviour).

### Objectives

The overall goal of this study is to design and conduct a survey of 9-1-1 call takers in the province of Ontario to better understand the factors associated with the successful identification of cardiac arrest (including patients with agonal breathing) over the phone and subsequent administration of CPR instructions to the caller. The specific study objectives are:

1) To conduct iterative semi-structured interviews to identify behavioural factors influencing identification of cardiac arrest and administration of CPR instructions by 9-1-1 call takers;

2) To develop a survey instrument about behavioural factors influencing the ability of 9-1-1 call takers to identify cardiac arrest and administer CPR instructions based on a systematic review of the literature [33], the results of the semi-structured interviews, and theoretical constructs from the TPB; and

3) To conduct a survey among Ontario 9-1-1 call takers using the survey instrument, to identify factors and strategies that might be targeted by knowledge translation interventions.

## Methods/Design

### Study design and setting

We propose to take a multi-phase approach to develop, pilot-test, and administer a survey examining the factors associated with the successful recognition of cardiac arrest by 9-1-1 call takers in the province of Ontario, Canada. Research ethics approval has been obtained from The Ottawa Hospital Research Ethics Board (2008512-01H). This study has been registered with clinicaltrials.gov (NCT00848588).

**Figure 2:**
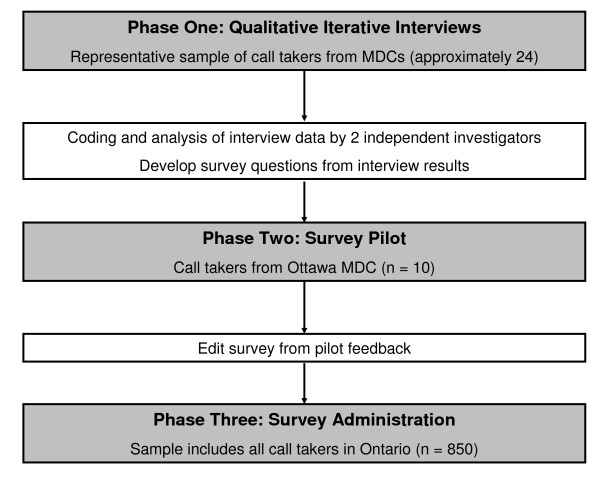
**Study flow**. MDC = medical dispatch centre.

The TPB methodology calls for a three-phase approach, which includes developing a survey from qualitative interview data (Phase One), a pilot test of the survey (Phase Two), followed by subsequent administration of the survey to the target population (Phase Three). See Figure 2 for study flow.

In Phase One of the project, we expect to conduct semi-structured interviews with 24 Ontario 9-1-1 call takers. The purpose of this phase is to identify and describe barriers and facilitators perceived to influence the ability of 9-1-1 call takers to recognise cardiac arrest (the target behaviour for this study) and give CPR instructions (the natural next step once cardiac arrest is recognized). Qualitative data from the interviews will be transcribed and coded sequentially. Recruitment of call takers will be purposeful, with the goal of obtaining a mix of responses from call takers who are employed in rural and urban medical dispatch centres, and have various levels of experience and training background. Interviews will be conducted until data saturation has been reached. The interviews will be audio-taped, with the participant's consent, and are expected to take approximately one hour. The participants will be offered an honorarium of $50 in recognition of the time required to participate. The data from this preliminary work will be used to inform the content of the quantitative survey.

In the survey development phase of the project (Phase Two), the data generated from the interviews will be used to develop and pilot test a quantitative survey examining the target behaviour, which is recognition of cardiac arrest by call takers. The survey will be organized using the theoretical constructs of the TPB which measure: behavioural intentions, attitudes, subjective norms, and perceived behavioural control. The initial draft of the survey will be circulated around the extended project team to ensure face and content validity. The survey will be piloted with approximately 10 call-takers from the Ottawa medical dispatch centre twice over a two-week period to ensure clarity and acceptability and to establish test-retest reliability. Data from pilot testing will be analyzed for temporal stability and internal consistency using standard techniques [34].

In Phase Three of the project, we plan to use a modified Dillman technique for the distribution of the survey [35]. An initial electronic notification about the survey will be sent to all identified call takers. All incorrect e-mail addresses will be noted and attempts will be made to identify the correct address. One week later, the survey will be sent to the call takers electronically. A reminder e-mail will be sent to all non-responders two weeks after the initial survey was sent. The survey will be administered using an electronic medium [36]. The invitation emails will contain a link to the survey website. However, respondents will be given the option to save their responses and finish the survey at a later time, if they are not able to complete the survey in one sitting. For all non-responders, two weeks following the reminder e-mail, a paper version of the survey will be mailed, with addressed stamped envelopes. The data from the paper surveys will be reconciled with the data from the electronic surveys.

### Study population

There are currently 23 medical dispatch centres in Ontario that employ approximately 850 call takers [personal communication, Ontario Ministry of Health and Long-Term Care, [8]. We will approach managers of the selected medical dispatch centres and ask for their assistance in recruiting call takers to participate in Phase One (iterative surveys) and Phase Three (survey). In Phase Three, the finalized survey will be circulated to our target population – all 9-1-1 call takers in the province of Ontario.

### Sample Size

Power calculations for multiple regression analysis of Phase Three survey will depend on the number of cases per predictor variable. A minimum sample size of 50 + 8 m, where m is the number of predictor variables, is recommended for testing the multiple correlation, and 100 + 8 m for testing individual predictors [37,38]. The survey will be organized using the theoretical constructs of the theory of planned behaviour which measure: behavioural intentions, attitudes, subjective norms, and perceived behavioural control. Assessments for each of the four theoretical constructs for both behaviours under study will include direct and indirect belief-based measures; each measure will use a minimum of three items on a 7-point Likert scale. Our survey should measure approximately 10–12 items, requiring a minimum sample size of 146 to test the multiple correlation, or 196 to test individual predictors.

### Methods of measurement

For Phase One, the audio-tapes of the interviews will be transcribed verbatim and verified by the interviewer prior to analysis. Data will be analyzed to identify themes and codes, including intentions to perform the behaviour, attitudes, subjective norms and perceived behavioural control of the behaviour. Two researchers will independently analyse the content of the responses, identify themes, and list them in order of frequency [39-42].

In Phase Two and Three, the survey data will be entered into a secure database developed by the Data Methods Centre at the Ottawa Hospital Research Institute. Upper and lower limits will be set for each variable and logical and range errors will be detected immediately by the program and highlighted for correction. Ten percent of case records, randomly selected, will be re-entered to assess data entry accuracy.

### Data analyses

Based on standard methodologies for developing measures of the intention, attitudes, subjective norm, and perceived behavioural control proposed in the TPB, [25,26,34,43,44] content analysis of the qualitative data from Phase One will identify: 1) the most frequently perceived advantages and disadvantages of recognizing cardiac arrest and administering CPR instructions to the caller (behavioural beliefs); 2) the most important people or groups of people who would influence the motivation of a 9-1-1 call taker to recognize cardiac arrest and give CPR instructions (normative beliefs); and 3) whether 9-1-1 call takers feel it is within their control to recognize cardiac arrest over the phone and give CPR instructions (control beliefs).

The planned analyses for Phase Two and Three will involve examining whether the constructs making up the TPB are significantly related to our primary outcome, specifically: whether attitudes, subjective norms and perceived behavioural control are related to the 9-1-1 call taker's intention and ability to recognize cardiac arrest over the phone and administer CPR instructions. Analysis of the survey data will include descriptive statistics for the most commonly cited barriers and facilitators of our target behaviour. Analysis of the hypotheses will be carried out by blocked multiple regression in order to test the strength of the relationship of these outcomes with the constructs predicted by the TPB. In addition to standard regression modeling, we will also produce a structural equation model of our data which will allow us to model not only direct relations between constructs and outcomes, but also indirect relationships through intervening constructs. This technique is useful for testing the validity of whole theories within a single analysis.

## Discussion

This study will address important knowledge gaps in the understanding of the barriers and facilitators experienced or perceived by 9-1-1 call takers in successfully recognizing cardiac arrest when agonal breathing is present. A review of the current literature suggests that the ability of 9-1-1 call takers to identify agonal breathing and cardiac arrest can be greatly improved [33]. What is not clear is how the 9-1-1 call taker's intervention can be improved to benefit a larger proportion of cardiac arrest victims. It is anticipated that the survey will identify key areas where improvements can be made in the training of 9-1-1 call takers or in the protocols they currently use for identification of cardiac arrest. We estimate that 9-1-1 assisted CPR instructions can, at the moment, contribute to saving as many as 360 lives annually in Canada. We plan to apply the findings of this pilot project in a future interventional project to test new interventions in order to increase the number of cardiac arrest victims receiving early bystander CPR and ultimately save more lives.

We are hopeful that our findings will make a significant impact at the occasion of the next iteration of the International Guidelines on Emergency Cardiovascular Care. These guidelines will be published by the American Heart Association in collaboration with the Heart and Stroke Foundation of Canada in 2010 [45]. We also intend to lobby the government at the municipal, provincial, and national level for the adoption of these improved delivery strategies for dispatch-assisted CPR instructions.

Ultimately, the most reliable measure of impact from all proposed interventions will be an unequivocal increase in bystander and survival rates for out-of-hospital cardiac arrest victims.

## List of abbreviations used

OOHCA: out-of-hospital cardiac arrest; CPR: cardiopulmonary resuscitation; EMS: emergency medical services; TPB: Theory of Planned Behaviour

## Competing interests

The authors declare that they have no competing interests.

## Authors' contributions

CV and MC conceived the study, obtained ethics approval, and funding. JLJ helped draft and edit the manuscript. CV, JLJ and AK developed the Phase One interview guide. CV, JLJ and AK helped coordinate the operational aspects of the study. JG, JB, GW and IS assisted with the methodology and revised it critically for important intellectual content. All authors read and approved the final manuscript.

## Pre-publication history

The pre-publication history for this paper can be accessed here:


